# The role of hippocampal glial glutamate transporter (GLT‐1) in morphine‐induced behavioral responses

**DOI:** 10.1002/brb3.2323

**Published:** 2021-08-07

**Authors:** Negin Saeedi, Mahgol Darvishmolla, Zohreh Tavassoli, Shima Davoudi, Soomaayeh Heysieattalab, Narges Hosseinmardi, Mahyar Janahmadi, Gila Behzadi

**Affiliations:** ^1^ Department of Physiology Medical School Shahid Beheshti University of Medical Sciences Tehran Iran; ^2^ Neuroscience Research Center Shahid Beheshti University of Medical Sciences Tehran Iran; ^3^ Division of Cognitive Neuroscience University of Tabriz Tabriz Iran

**Keywords:** dependence, glial cells, glutamate transporter‐1, hippocampus

## Abstract

Opioid abuse modifies synaptic plasticity, which leads to behavioral changes, such as morphine dependence, but the mechanism remains poorly understood. Glial cells play an important role in the modulation of synaptic plasticity and are involved in addictive‐like behaviors. The indisputable role of glutamate in opiate addiction has been shown. Astrocytes, a type of glial cells, which are integral functional elements of synapses, modulate the concentration of glutamate in the synaptic space. One of the most important mechanisms for glutamate concentration regulation is its uptake from the synaptic cleft. In this study, we evaluated the role of hippocampal glial glutamate transporter (GLT‐1) in morphine dependence. Male rats received subcutaneous (s.c.) morphine sulfate (10 mg/kg) at an interval of 12 h for 9 days. In order to activate GLT‐1, animals received an intrahippocampal injection of ceftriaxone (0.5 mmol/0.5 μl) in the CA1 region of the hippocampus, 30 min before each morphine administration. Rats were assessed for morphine dependence by monitoring naloxone hydrochloride‐induced morphine withdrawal. Our results showed that hippocampal microinjection of ceftriaxone, as an activator of GLT‐1, reduced some signs of morphine withdrawal, such as activity, diarrhea, head tremor, freezing, and ptosis. It seems that hippocampal GLT‐1 can be affected by chronic morphine administration and involved in morphine dependence. Therefore, its activation may reduce morphine side effects by reducing hippocampal glutamate.

## Graphical abstract

Hippocampal GLT‐1 and morphine‐dependence.

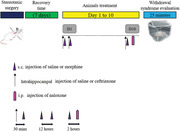

## 1 INTRODUCTION

Drug addiction is considered to be a chronic and recursive neurological disorder. Opioids are commonly misused classes of drugs that are usually prescribed to relief chronic and acute pain. Chronic exposure to opioids produces complex and permanent changes in the brain (Morgan & Christie, 2011). One of the most commonly used opioids that has received less attention in studies is morphine. Long‐term use of morphine causes dependence and tolerance to its analgesic effects. High similarities between addiction and learning and memory mechanisms and creating false memories following chronic morphine administration have been postulated (Torregrossa & Corlett, 2011). One of the factors that play a key role in memory and learning mechanism is glutamate, which is a stimulatory neurotransmitter in the brain. Since addiction is also a type of ectopic memory, this stimulatory neurotransmitter has an important role to play in addiction (Aguilar et al., 2009). For example, among the criteria that have been suggested to cause dependence is an imbalance of glutamate and GABA as excitatory and inhibitory neurotransmitters, respectively (Banerjee, 2014). In this regard, many studies have proposed the role of N‐methyl‐D‐aspartate (NMDA) receptors in the development of dependence (Tomek & LaCrosse, 2013). Studies also demonstrate that glutamate is one of the neurotransmitters involved in relapse to drug‐seeking so that injection of NMDA receptor antagonists inhibits morphine relapse (Peters & De Vries, 2012). Therefore, maintenance of glutamate homeostasis seems to be of particular importance, and changes in its extracellular concentration have been reported during chronic drug abuse (Wang & Zeng, 2019). Studies have shown that the concentration of glutamate in the brain increased after chronic morphine administration (Kemppainen & Nurmi, 2015). This increase is associated with dependence and withdrawal syndrome. Glutamate plays a key role in neural circuits, which perform specific functions like formation and retrieval of memory (Basu, 2015). Previous studies have shown that drug abuse affects reward circuits and related structures, such as the hippocampus (Cooper & Robison, 2017).

In addition to neurons, glial cells, especially astrocytes, are involved in glutamate release and uptake and also in side effects of chronic opioid exposure, including tolerance and dependence (Mahmoud & Gharagozloo, 2019; Miguel‐Hidalgo, 2009). Our previous studies reported that glial cells in the hippocampus may play role in dependence (Seyedaghamiri & Heysieattalab, 2018). Besides the presence of morphine receptors on neurons, the mu‐opioid receptor which seems to mediate the euphoric effects of opioids, is widely expressed in hippocampal astrocytes (Nam & Han, 2018) Therefore, glial cells appear to be involved in morphine‐induced metaplasticity and consequent behavioral changes. One of the important functions of glial cells that can be important in metaplasticity and addiction is changing the concentration of glutamate in the synaptic space.

Among the factors involved in glutamate homeostasis are glutamate transporters. Glutamate transporter‐1 (GLT‐1) is mainly expressed in astrocytes (Kobayashi & Nakano, 2018) and it makes up more than 80% of hippocampal glutamate transporters and removes 90% of extracellular glutamate (O'Donovan & Sullivan, 2017). As opioids alter the expression of GLT‐1, they may dysregulate glutamate homeostasis and ultimately lead to drug‐seeking behaviors (Roberts‐Wolfe & Kalivas, 2015). Studies have demonstrated that stimulation of GLT‐1 expression and function by beta‐lactam antibiotics, such as ceftriaxone, reduced drug‐induced reinstatement of methamphetamine and nicotine conditioned place preference (Alajaji & Bowers, 2013). Repeated ceftriaxone treatment reduces alcohol drinking in male and female rats coincident with an increase in GLT‐1 levels in prefrontal cortex and NAc (Sari & Toalston, 2016).

Considering the synaptic alterations after long periods of opioid exposure in the hippocampus, and the involvement of glial cells in opioid addictive behaviors, and regarding the GLT‐1 downregulation following chronic morphine administration, we aimed to investigate the role of the glutamate transporters of hippocampal glial cells in morphine dependence. Finding the exact mechanisms involved in morphine addiction can be important to prevent its side effects. Determining the different functions of glial cells and targeting them as non‐neuronal cells could be appropriate in preventing or treatment of morphine addiction.

## 2 MATERIALS AND METHODS

### 2.1 Animals

Adult male Wistar rats weighing 200–250 g were used in this study. They were housed in an animal room with a controlled temperature (22 ± 1°C) under 12 h light/dark cycle (lights on 07:00 am). All experiments were performed from 09:00 am to 12:00 am. Food and water were available ad libitum. Rats were housed three per cages, but after surgery, they were kept in individual cages. All experimental procedures were conducted following the policy of the Iranian Convention for the Protection of Vertebrate Animals Used for Experimental Purposes and they were approved by the Ethics Committee of Shahid Beheshti University of Medical Sciences, Tehran, Iran.

### 2.2 Drugs

Drugs used in this study are as follows: Morphine Sulfate (Temad, Tehran, Iran), Naloxone Hydrochloride (Sigma‐Aldrich, St. Louis, MO, USA), and ceftriaxone disodium salt hemi (Heptahydrate, Sigma‐Aldrich). Morphine and ceftriaxone were dissolved in sterile 0.9% saline before each experiment.

### 2.3 Surgical procedures and microinjection of drugs

**FIGURE 1:**
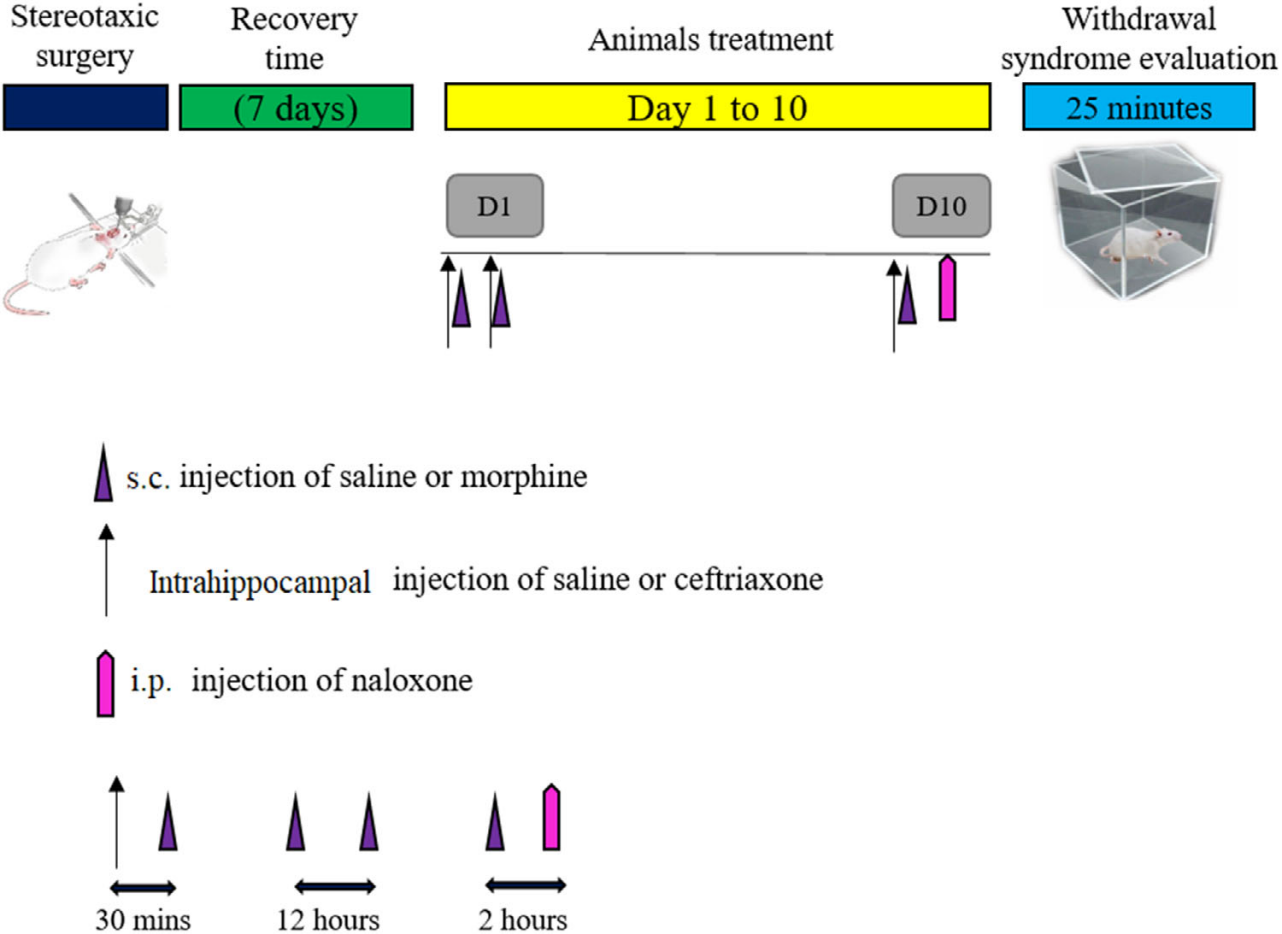
Experimental protocol of the study

**FIGURE 2:**
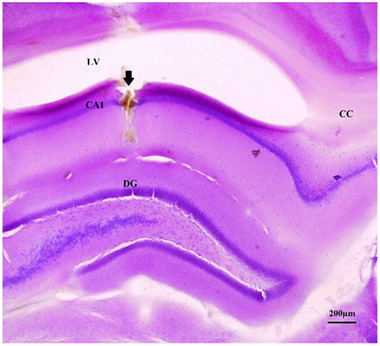
Representative cresyl violet‐stained coronal brain section shows cannula placement in the CA1 area of dorsal hippocampus (arrow). Abbreviations: CA1, field CA1 of Ammons horn; CC, corpus callosum; DG, dentate gyrus; LV, lateral ventricle

Rats were anesthetized with an injection of Xylazine 2% (10 mg/kg) and Ketamin 10% (100 mg/kg). They were fixed in the stereotaxic device (Stoelting Instruments, Wood Dale, IL, USA). The injection site in CA1 area of the hippocampus was bilaterally marked with coordinates (AP = −2.8 mm posterior to the bregma; ML = ±1.8 mm lateral to the midline; DV = 2.5−3.5 mm, Paxinos & Watson, 2006). Guide cannulae were then implanted into marked site for injection of ceftriaxone (Figure 2). The cannula was a 22‐gauge stainless steel, which was secured with dental cement. In order to prevent the cannula from coming out of the skull, the surface was thoroughly dried of blood before placing the cement on the skull. Fixing a screw to the skull was also used to help stabilize the cannulas. Moreover, in order to prevent the impairment of CA1 tissue, the cannulae was inserted very slowly and after each experiment, the brain was removed and the extent of the lesion was checked. As shown in Figure 1, the injections were started 7 days after the cannula implantation to allow the animals to recover from surgery. Before morphine injection (s.c., 10 mg/kg), ceftriaxone was microinjected bilaterally into the CA1 region with an interval of 12 h (30 min before each morphine injection) for 9 days. A polyethylene tube was attached to a 1 μl Hamilton syringe for injection via cannula. Intra‐CA1 infusions of ceftriaxone or its vehicle were carried out in each side over a period of 60 s and it kept at the injection site for 30 s to ensure the accuracy of the injection.

### 2.4 Induction of morphine dependence and naloxone‐precipitated withdrawal syndrome

After the last injection of morphine, on the 10th day, naloxone hydrochloride (1.5 mg/kg) was administered intraperitoneally in order to induce morphine withdrawal syndrome. Rats were placed in a Plexiglas cylinder test chamber (30 cm diameter, 50 cm height) and their behaviors were monitored for 25 min. An investigator, who is blind to the treatment condition, monitored the withdrawal signs. The protocol of the experiment is presented in Figure 1.

In groups 1 and 2, animals received a bilateral intrahippocampal infusion of saline (0.5 μl) before each subcutaneous saline or morphine (10 mg/kg, s.c.) injection every 12 h for 9 days (12 rats/group).

In order to investigate the role of hippocampal GLT‐1 in morphine dependence, animals in group 3 received a bilateral intrahippocampal infusion of ceftriaxone (0.5 mmol/0.5 μl), as a GLT‐1 activator, before each morphine injection (9 rats/group).

To evaluate the effect of hippocampal GLT‐1 activation on naloxone‐precipitated responses in nondependent rats, in another group (group 4), ceftriaxone (0.5 mmol/0.5 μl) was microinjected into the hippocampus before each saline injection (9 rats/group).

In all groups, naloxone (1.5 mg/kg, i.p.), an opioid antagonist, was injected to precipitate withdrawal syndrome in order to assess the morphine dependence on the 10th day.

### 2.5 Cannula verification

At the end of each experiment, animals were anesthetized with CO2 and the brains were extracted and cut by vibroslicer (Campden Instrument, NVSLM1, Sarasota, FL, USA) in coronal 50 μm sections to verify the cannula placement. An image of cresyl violet‐stained showing injection site in CA1 area is demonstrated in Figure 2. Data from animals with misplaced cannula were not included in the statistical analysis and replaced by new ones to reach the standard sample size.

### 2.6 Data analysis

Data were analyzed using GraphPad Prism 6.0 (GraphPad, La Jolla, CA, USA). Data collection and analysis were done blind. The Kolmogorov–Smirnov test (K–S test) was used for the analysis of normal distribution. All comparisons were initially subjected to one‐way ANOVA. Post hoc analysis was done by Tukey's multiple comparisons test. Kruskal–Wallis test was used in the absence of normal distribution of the data and post hoc analysis was done by Dunn's multiple comparisons test. Results were presented as mean ± SEM when data distribution was symmetrical. Otherwise, results were expressed as the median and interquartile range (IQR). *p* < .05 was considered statistically significant.

## 3 RESULTS

**FIGURE 3:**
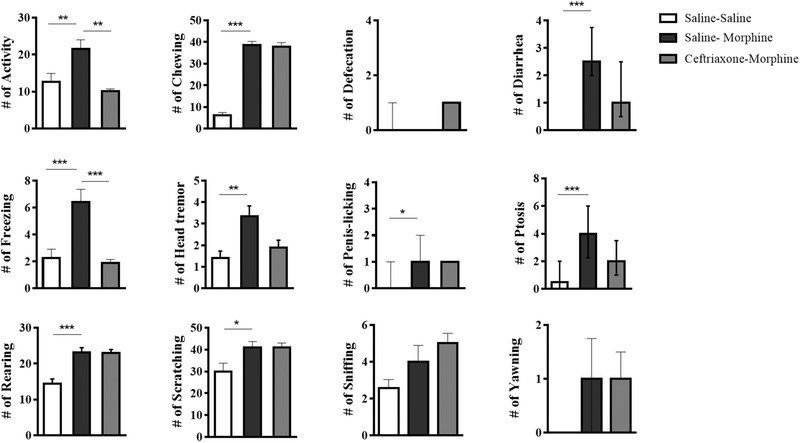
Naloxone‐precipitated behavioral responses in morphine‐dependent and nondependent rats with or without ceftriaxone microinjection. Symptoms of withdrawal syndrome in animals that received s.c. morphine and intrahippocampal saline (group 2; saline‐morphine, *n* = 12) were demonstrated compared to the group that received s.c. and intrahippocampal saline (group 1; saline‐saline, *n* = 12). Signs of withdrawal in animals that received intrahippocampal ceftriaxone pretreatment before each injection of s.c. morphine (group 3; ceftriaxone‐morphine, *n* = 9) were shown compared to the group 2. All data represent mean ± SEM except defecation, diarrhea, penis licking, ptosis, and yawning, which is expressed as median and interquartile range (IQR). One‐way ANOVA followed by Tukey and Dunn's post hoc test in parametric and nonparametric data, respectively. *p < .05, **p < .01, ***p < .001

To induce morphine dependence, 10 mg/kg morphine was injected subcutaneously every 12 h for 9 days, and 1.5 mg/kg naloxone was injected on the 10th day to induce withdrawal syndrome. There were significant differences between groups in all withdrawal symptoms, including activity, chewing, diarrhea, freezing, rearing (*p* = .0001), sniffing (*p* = .02), head tremor (*p* = .01), penis licking (*p* = .008), scratching (*p* = .02), ptosis (*p* = .001), and yawning (*p* = .04) except defecation. Post hoc analysis showed that there were significant differences between morphine‐dependent animals (group 2, *n* = 12) and saline‐treated (nondependent, group 1, *n* = 12) rats in activity: [*F* (3, 38) = 9.1, *p* = .001], chewing: [*F* (3, 38) = 119.4, *p* < .0001], freezing: [*F* (3, 37) = 9.43, *p* < .0001], head tremor: [*F* (3, 38) = 4.33, *p* = .0101], rearing: [*F* (3, 38) = 18.09, *p* < .0001], scratching: [*F* (3, 38) = 3.56, *p* = .02] (one‐way ANOVA, mean ± SEM), diarrhea: [*H* (3) = 30.78, *p* < .0001], penis licking [*H* (3) = 11.83, *p* = .008], and ptosis [*H* (3) = 15.50, *p* = .001] (one‐way ANOVA, median ± IQR, Figure 3).

To evaluate the involvement of hippocampal glutamate transporter‐1 in morphine dependence, 30 min before each injection of morphine, hippocampal GLT‐1 was activated by ceftriaxone infusion (0.5 μl of 0.5 mM solution) into the CA1.

Post hoc analysis demonstrated that some withdrawal symptoms, including activity, freezing, head tremor (one‐way ANOVA, mean ± SEM), ptosis, and diarrhea (one‐way ANOVA, median ± IQR), decreased in the morphine‐treated group receiving ceftriaxone before each morphine (group 3, *n* = 9), compared with the morphine‐dependent group receiving saline (group 2, *n* = 12, Figure 3).

Moreover, ceftriaxone vehicle (saline) microinjection into the hippocampus did not affect the naloxone‐precipitated morphine withdrawal signs in morphine‐dependent rats. The animals that received the saline before morphine injection were exhibited withdrawal signs following naloxone administration, which may suggest that the effects of ceftriaxone were probably due to a pharmacological effect.

**FIGURE 4:**
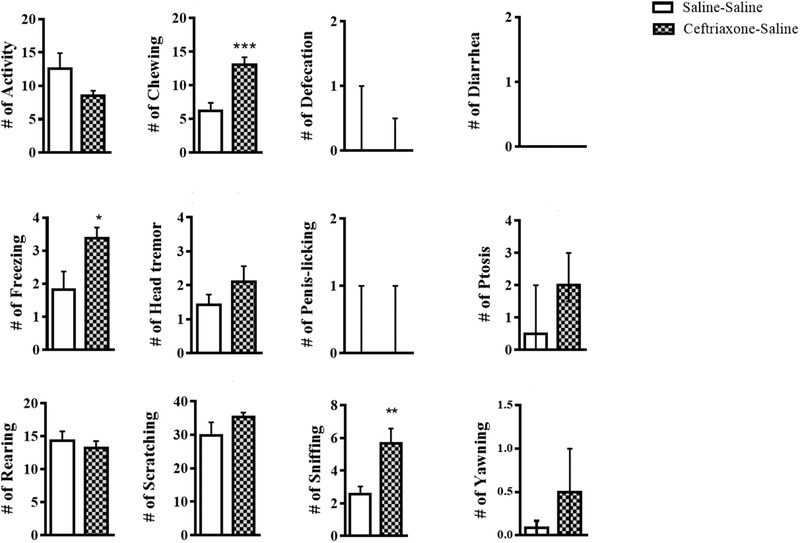
Naloxone‐precipitated behavioral responses in nondependent rats with or without ceftriaxone microinjection. Symptoms of withdrawal like syndrome in nondependent animals receiving ceftriaxone (*n* = 9) are shown in comparison with the group receiving saline (*n* = 12). All data represent mean±SEM, except ptosis, yawning, diarrhea, defecation, and penis‐licking that is expressed as median and interquartile range (IQR). Unpaired *t*‐test or Mann–Whitney U test, * *p* < .05, **p < .01, ***p < .001

Activation of hippocampal GLT‐1 in nondependent animals (group 4, *n* = 9) did not cause withdrawal‐like symptoms. Comparing the behavior of animals receiving saline in the hippocampus (group 1, *n* = 12) with animals receiving ceftriaxone in the hippocampus (group 4, *n* = 9) did not differ significantly in response to naloxone injection (unpaired *t*‐test or Mann–Whitney U test, *p* > .05, Figure 4). Injection of ceftriaxone in the hippocampus in nondependent animals just increased chewing, freezing, and sniffing (unpaired *t*‐test, *p* < .05, Figure 4).

## 4 DISCUSSION

The results of this study indicate that activation of GLT‐1 by ceftriaxone in the CA1 region of the hippocampus during chronic administration of morphine decreases some symptoms of naloxone‐induced withdrawal syndrome.

This study aimed to investigate the effect of ceftriaxone‐mediated activation of GLT‐1 in the hippocampal CA1 region on morphine dependence. Ceftriaxone is a β‐lactam antibiotic that enhances the expression and activity of GLT‐1 in astrocytes (Rothstein & Patel, 2005). Ramos et al. showed that intrathecal injection of ceftriaxone significantly increased spinal GLT‐1 protein expression in male rats (Ramos & Lewis, 2010). Considering the confirmed concentration to increase GLT‐1 expression and activity, in the present study, ceftriaxone (0.5 mmol/0.5 μl) was used as a tool to activate hippocampal glial glutamate transporter.

According to our data, activation of glutamate transporter reduced withdrawal symptoms. Disturbing the balance between glutamate and GABA can contribute to addictive behaviors (Samardzic & Jadzic, 2018). Studies have shown that addictive substances can change the concentration of glutamate in the synaptic space. For instance, chronic administration of morphine increases the concentration of glutamate in the synaptic space (Chen, 2015). Therefore, in our study, ceftriaxone by activating GLT‐1 may prevent the increase in glutamate concentration due to morphine. So, it reduced morphine dependence and withdrawal signs. As studies have found that NMDA receptor and AMPA (α‐amino‐3‐hydroxy‐5‐methyl‐4‐isoxazolepropionic acid) receptor antagonists during chronic exposure to opioids block the progression of dependence and withdrawal syndrome (Van Sickle & Xiang, 2004), our study emphasized the glutamate role in morphine dependence. As mentioned, the reduction in glutamate concentration by ceftriaxone administration led to decrease in morphine dependence and withdrawal syndrome.

In addition to the neurons, glial cells, especially astrocytes, play an important role in regulating the release and uptake of various gliotransmitters, including glutamate (Mahmoud & Gharagozloo, 2019). Interestingly, various studies have shown that the use of drugs, such as morphine, amphetamine, and cocaine, activates astrocytes in the hippocampus, spinal cord, periaqueductal gray, and posterior cingulate cortex (Hao & Liu, 2011; Miguel‐Hidalgo, 2009 ). More interestingly, increased activity of these cells by specific mechanisms seems to mediate the rewarding effects of substance abuse. Therefore, it seems that manipulation of glial cells can cause changes in dependence and withdrawal symptoms. (Narita & Miyatake, 2006). As noted above, besides neurons, glial cells can actively release and uptake glutamate. Glutamate transporters, like GLT‐1, are among the factors involved in regulating glutamate and are needed to maintain glutamate homeostasis in the brain (Passlick & Rose, 2021). It can be deduced that opioids affect GLT‐1 expression and glutamate removal from the synaptic space (Scofield & Kalivas, 2014). Rawls et al. showed that morphine administration and withdrawal induction increase glutamate uptake and GLT‐1 expression in hippocampal synaptosomes. Based on this study, glutamate reduction in the synaptic space following chronic administration of morphine is expected (Rawls & Zielinski, 2010). A study by Nakagawa and Satoh showed increased extracellular glutamate concentrations in locus coeruleus following discontinuation of morphine administration (Nakagawa & Satoh, 2004). Our behavioral results parallel with Nakagawa and Satoh showed that by activating GLT‐1, morphine dependency can decrease. Consistent with our results, Ozawa et al. (Ozawa & Nakagawa, 2004) found that increased expression of GLT‐1 in locus coeruleus prior to morphine injection inhibited naloxone‐induced withdrawal symptoms, and GLT‐1 may have an inhibitory role in morphine dependence. Similarly, other studies observed a decrease in behavioral responses, such as nicotine‐seeking behavior following the activation of GLT‐1, and also a decrease in extracellular glutamate concentration (Alajaji & Bowers, 2013).

As shown in the results, some withdrawal signs reduced greater than others by hippocampal GLT‐1 activation. Given the density of GLT‐1 in the hippocampus (Heo & Jung, 2012) and the role of the hippocampus in memory and learning, it is likely that the involvement of the hippocampus in different aspects of dependence and withdrawal symptoms may be different. Another probability is that producing different aspects of morphine dependence and withdrawal symptoms needs different concentrations of glutamate.

What has been discussed thus far is related to the regulation of glutamate concentration by GLT‐1 in the extracellular space. However, extracellular glutamate concentration depends on several factors. For example, glutamate levels differ in resting conditions and lack of action potential. The concentration of this neurotransmitter also depends on the interaction between glial cells and neurons by removal from the synaptic space by mGluR, AMPA, and NMDA receptors (Kalivas, 2011). Perhaps, because we did not see any decrease in symptoms of withdrawal syndrome following GLT‐1 activity, there are other adaptive mechanisms that, despite GLT‐1 activity, were able to maintain elevated glutamate levels in the hippocampal synaptic space due to morphine consumption.

On the other hand, the brain's reward system is broad, encompassing various areas, including the hypothalamus, Ventral Tegmental Area (VTA), Nucleus Accumbens (NAc), amygdala, locus coeruleus, and hippocampus (Kim & Ham, 2016). Glutamatergic neurons in the CA3 region of the hippocampus activate the GABAergic nucleus accumbens neurons, which increase dopamine release in the VTA by inhibiting the inhibition of GABAergic translocation to the VTA (Luo & Tahsili‐Fahadan, 2011). As our obtained data show, manipulating the hippocampus changed some symptoms greater than others. It emphasized that the role of different circuits of the reward system may be varied in the occurrence of different properties of addiction. The symptoms of morphine withdrawal are also very widespread, and each can functionally affect a specific area of the brain and circuits (Kim & Ham, 2016; Meye & Trusel, 2017 ). Therefore, due to different circuits involved in different dimensions of dependence and different abstinence behaviors, and different mechanisms in regulating extracellular glutamate concentration, in this study, some symptoms of withdrawal syndrome have been reduced by activating glutamate transporter of the hippocampal glial cells.

Interestingly, ceftriaxone administration in nondependent animals increased some symptoms of withdrawal syndrome. As mentioned above, activation of the glutamate transporter by ceftriaxone decreases the concentration of glutamate in the synaptic space (Chotibut & Davis, 2014). Studies have also reported a direct relationship between glutamate concentration and endogenous opioids. It has been found that more repetitive behaviors are observed when glutamate and endogenous opioids decrease (Augustine & Rajendran, 2020). It seems that in our study, endogenous opioids have been diminished by reducing glutamate concentration due to GLT1 activation. Therefore, when naloxone was injected in nondependent animals, withdrawal‐like symptoms were observed.

Therefore, it may be concluded that hippocampal glial glutamate transporters are involved in behavioral changes due to morphine exposure. Neural–glial interactions influence addictive behavioral responses by several ways. Based on the present study, it can be concluded that one of these mechanisms is the regulation of hippocampal synaptic glutamate concentration by GLT‐1. Further investigations are needed to elucidate the other mechanisms of glial involvement in development and maintaining addictive behaviors.

### PEER REVIEW

The peer review history for this article is available at https://publons.com/publon/10.1002/brb3.2323
